# A primer in artificial intelligence in cardiovascular medicine

**DOI:** 10.1007/s12471-019-1286-6

**Published:** 2019-05-20

**Authors:** J. W. Benjamins, T. Hendriks, J. Knuuti, L. E. Juarez-Orozco, P. van der Harst

**Affiliations:** 1 University of Groningen, University Medical Center Groningen, Department of Cardiology, Groningen, The Netherlands; 2 Turku PET Center, Turku University Hospital and University of Turku, Turku, Finland; 3 Durrer Center for Cardiovascular Research, Netherlands Heart Institute, Utrecht, The Netherlands; 4 University of Groningen, University Medical Center Groningen, Department of Genetics, Groningen, The Netherlands

**Keywords:** Artificial intelligence, Machine learning, Deep learning, Artificial neural networks, Cardiovascular disease

## Abstract

Driven by recent developments in computational power, algorithms and web-based storage resources, machine learning (ML)-based artificial intelligence (AI) has quickly gained ground as the solution for many technological and societal challenges. AI education has become very popular and is oversubscribed at Dutch universities. Major investments were made in 2018 to develop and build the first AI-driven hospitals to improve patient care and reduce healthcare costs. AI has the potential to greatly enhance traditional statistical analyses in many domains and has been demonstrated to allow the discovery of ‘hidden’ information in highly complex datasets. As such, AI can also be of significant value in the diagnosis and treatment of cardiovascular disease, and the first applications of AI in the cardiovascular field are promising. However, many professionals in the cardiovascular field involved in patient care, education or science are unaware of the basics behind AI and the existing and expected applications in their field. In this review, we aim to introduce the broad cardiovascular community to the basics of modern ML-based AI and explain several of the commonly used algorithms. We also summarise their initial and future applications relevant to the cardiovascular field.

## Introduction

The recent resurgence of artificial intelligence (AI) and its implementation in a wide range of industrial and scientific areas has attracted the attention of clinicians. The confluence of unprecedented computational power and data storage capacity has galvanised the deployment and refinement of AI and especially the subdomain of machine learning (ML) algorithms in prevention, diagnostics, risk stratification, and treatment selection tasks in medicine. After successful proof-of-concept implementations of ML-based AI in areas such as dermatology [1] and ophthalmology [2], the advancement of these methods has steadily reached the field of cardiology. To understand the relevance and potential of this AI explosion in cardiology, the present review aims to provide a primer on the essentials of modern ML-based AI, and to summarise the most recent AI implementations in cardiovascular research as well as its initial clinical applications. Finally, we will present a succinct view into future perspectives in the field.

### Historical context

The term AI was first coined by a group of pioneers at a conference held in 1956 at Dartmouth College in New Hampshire, USA [3]. Their goal was to develop computer systems able to perform tasks that normally require human intelligence. The US Department of Defense invested greatly in several projects, which were ultimately terminated due to underestimated complexity and lack of computational power [4, 5].

**Fig. 1:**
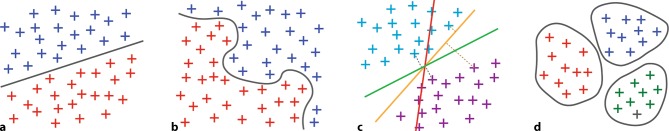
**a**–**d** Different approaches to data separation in classification problems. For this illustrative purpose, the presented data exist in 2D space. Dimensionality in real datasets and images is much higher, but the same principles apply. **a** Example of two classes of samples that can be separated linearly. **b** Two non-linearly separable data classes. **c** Data separation in a support vector machine. The optimal support vector (*green*) keeps a balanced optimal distance to all data in both classes to prevent misclassification in added data points.** d** Example of multiple data clusters. The samples in each cluster belong together, based on their relatively close distances

As a subdomain in the field of AI conceived in 1959, ML uses mathematical methods to make predictions on or classifications of data, without the need for programmed rules and prior knowledge by human beings. Initial ML approaches were developed to resolve simple, linear problems (Fig. 1a). In the following decades, artificial neural networks were developed, functioning over a cascade of multiple processing units and providing the ability to solve highly complex, non-linear problems (Fig. 1b).

Initially evolving relatively anonymously, the field of AI gained widespread popularity after the first artificial neural network won the 2012 ImageNet computer vision competition [6]. Further growth was driven by the development of graphical processing units (GPUs) developed for the gaming industry. These processors are capable of greatly enhancing parallel processing of large amounts of data, which allows for training of very complex models in large amounts of data.

**Fig. 2:**
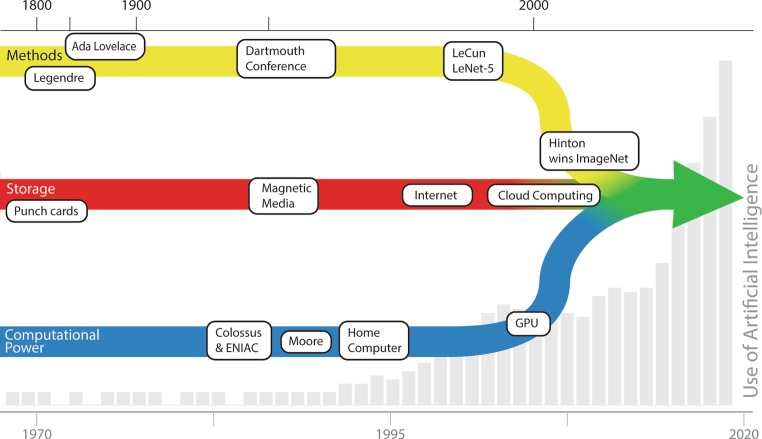
Various influences on the development of deep learning. *Methods*: Starting with Legendre, mathematicians have developed various techniques, some of them specifically aimed at artificial intelligence since the Dartmouth conference. LeCun developed the convolutional neural network (1989), used by Hinton to win the 2012 ImageNet competition. *Storage*: Maturing over a century, grown significantly after the introduction of the internet, leading to current cloud systems. *Computational power*: After the introduction of computers during World War II, an exponential growth of computing power was recognised by Moore, continuing until today. Driven by the gaming industry, graphical processing units have given computers the final boost to implement deep learning

Parallel to this development, the rise of cloud platforms that provide highly scalable processing power and the ability to store large amounts of data required to train such algorithms has boosted this progress. Cumulating a cascade of related and unrelated historic, societal and scientific developments, the current surge of AI implementations is an obvious and irreversible event (Fig. 2).

Dutch universities recently reported student registration for their AI programmes to far exceed their maximum capacity [7], the Massachusetts Institute of Technology has announced a $1 billion investment in a new department with dedicated focus on AI [8], and the Japanese government has announced a $100 million investment to establish 10 AI hospitals [9]. AI is here to stay and will affect all aspects of science and society.

## Machine learning algorithms

### Concepts and terminology

AI refers to the broad concept of simulating human intelligence and its application to specific practical purposes, while ML pertains to the family of algorithms that share a capacity to iteratively elucidate patterns (i.e. learn), to optimise tasks like prediction or classification. Modern ML-based AI differs from other methods, such as knowledge-based expert systems, in that it is able to learn from data and does not depend on explicitly programmed instructions or mathematical assumptions. There is supervised learning, in which models are trained based on already labelled data (the so-called ground truth) and are typically used to make predictions on new data. Unsupervised learning, on the other hand, infers structural relations and dependencies from data without any prior knowledge or constraints about it. These models are used primarily to gain new insights on data structure. In reinforcement learning, a model learns to make decisions by being rewarded for its actions in a certain situation.

The learnable parameters in an ML model are known as weights, which are estimated by minimising a loss function, a measure of the model’s error. Feature extraction is a method to build derived variables such as borders, edges, lines, corners or colourings in images, and frequency information in time signals. It is used for dimensionality reduction, i.e. a way to remove redundant information (noise) from data. In this regard, feature selection is analogous to stepwise variable inclusion in traditional linear models.

Data analysis through ML is commonly undertaken by allocating the largest portion of a dataset to a training set, which will be used as input for initiating the learning of the selected ML model. At the same time, a smaller portion of the dataset is allocated to the validation set, which is utilised to offer an evaluation of a model fit while optimising its settings between iterative rounds. The objective of these two sets is to track the learning progress of the model (training) and to gauge the likely generalisation that this learning will have to future new data (validation). Finally, a randomly selected proportion of the original dataset should have been reserved to provide an independent and unbiased evaluation of the performance of the final model. This is known as the test set, and it attests to the expected utility of the model in the real world as it did not contribute to the training or validation processes.

### Supervised machine learning

#### Regression

Regression is the most basic approach towards ML, in which the effect of one (univariate) or more (multivariable) input variables on a continuous outcome variable is expressed by a linear function. Using ML, regression can also be applied to more complex data, such as images, e.g. models that have been trained to predict fractional flow reserve (FFR) from coronary computed tomography angiography (CCTA) images [10].

#### Classification, object detection and segmentation

**Fig. 3:**
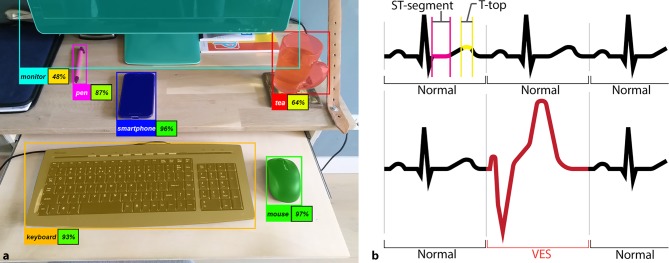
**a**, **b** Detection and segmentation of objects.** a** Detected objects are marked by a coloured rectangle and the identified object type. Segmentations are displayed by a semi-transparent colour overlay. A trained model may be more or less certain about its predictions, as indicated by the percentages alongside the object classifications. **b** Segmentation and object detection applied to an ECG signal. A trained model can detect separate segments (e.g. ST: *magenta*, T‑top: *yellow*) of the ECG and detect abnormal beats, such as a ventricular extrasystole (*VES*, *red*)

Classification is the process of identifying to which predefined category a sample belongs. A classic example is a model that is trained to recognise cats and dogs in images. Applied at a deeper level, object detection is used to classify subsets within the sample’s total data. A typical example is recognition of humans, animals or objects in a photograph (Fig. 3a), which are annotated by a rectangle, commonly referred to as bounding box [11]. In segmentation, an extended application of classification, structures in an image are partitioned into sets of pixels (Fig. 3a). Segmentation may also be applied to sequential, one-dimensional data, such as an electrocardiogram (ECG), and used for detecting intervals or abnormal beats (Fig. 3b).

#### Support vector machines

Support vector machines separate groups of samples based on a vector representation, by dividing them by a vector that keeps an optimal distance to samples in both classes (Fig. 1c). Support vector machines show resemblance to regression models, but can handle greater data complexity and are less susceptible to outliers.

#### Random forests

In random forest algorithms, a group of decision trees (forest) is trained to make decisions based on a range of variables and threshold values. These decision trees are similar to the diagnostic algorithms found in clinical guidelines, but usually more complex [12]. Their added value is that averaging over a group of decision trees generalises better to a wider range of input data.

### Unsupervised machine learning

#### Clustering

Clustering algorithms divide data into subgroups, called clusters, based on resemblances within the data. Data samples are treated as multidimensional vectors that are grouped in such a way that per cluster the average distance between all vectors and the cluster centre is minimised (Fig. 1d). The number of clusters is usually predefined by the user, although advanced software packages have been developed to automatically determine the optimal numbers of clusters [13–15].

#### Anomaly detection

Anomaly detection is specifically designed to find outliers in data that are related to the occurrence of an event that cannot simply be predicted using threshold values of one or more parameters. In healthcare, anomaly detection could be applied in intensive care units, to detect potential adverse events or signals for a required treatment based on outliers in measurements from different modalities [16].

### Deep learning

**Fig. 4:**
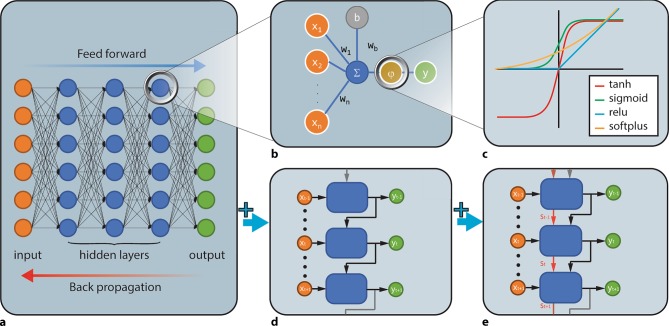
**a** Feedforward network. Input flows from left to right predict output values. **b** Artificial neuron: each input *x*_*i*_ is multiplied by its own weight *w*_*i*_. To introduce an intercept, input *b* (with value 1) is introduced, with its own weight *w*_*b*_. The sum of all weighed inputs *x* and intercept b is used as input for the activation function **φ** that yields an output above a certain threshold. **c** Examples of activation functions. *Tanh* is the hyperbolic tangent, with outputs quickly changing from −1 to 1 around input x = 0. The *sigmoid* resembles tanh, with outputs shifting from 0 to 1 around *x* = 0. *Relu* is the rectified linear unit, with output = 0 for inputs smaller than zero and outputs equal to input for inputs greater than or equal to zero. *Softplus* is a continuous function that only slightly differs from relu for inputs around zero and is described by ln(1 + *e*^*x*^) **d** Recurrent neural network, which adds a time component to the feedforward network. In addition to input *x*_*t*_ at a given moment *t*, the previous output y_t-1_ is passed through the model to predict y_t_. **e** Long short-term memory, which adds a persisted model state s in addition to the previous model output *y*_*t–*__1_, to better assess the effect of input changes, over a longer period, on model output

Deep learning (DL) is an important subset of ML methods that can be either supervised or unsupervised. DL has acquired major relevance due to its great success in image processing. The general structural design of DL models is known as a feedforward artificial neural network, an idea based on the physiological function of a neuron. Input data are transformed by subsequent layers of processing units to produce a predicted output (Fig. 4). In a typical neural network, each node is an activation function which can produce an output signal if the sum of the inputs exceeds a certain threshold level.

In supervised DL, training data are used as input to build a model over repeating training cycles. To optimise model performance, model weights are adjusted during each training round based on the differences between output and ground truth, each proportional to its relative contribution to the error. This process is in fact called error backpropagation. As soon as the ascending predictive performance reaches a plateau, the algorithm stops learning from new training data and the training process may be stopped.

In rare diseases, pathology can even be simulated to create artificial training data [17]. Interestingly, DL models are sometimes considered to be ‘black boxes’ and, in general, provide little insight into what they learn and how it was learnt. For this purpose, methodologies are currently being developed to visualise the learning process in DL [18].

#### Convolutional neural networks

**Fig. 5:**
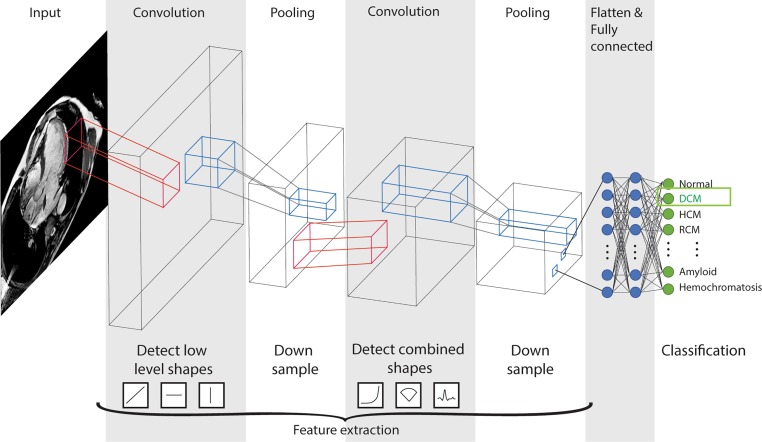
Schematic presentation of a convolutional neural network. Each convolutional layer develops several filters to detect shapes or objects in the previous layer (*red*), from basic shapes in the first convolutional layer to highly complex objects in the last layer. The thickness of a layer resembles its filter count. Following each convolutional layer, the data are down-sampled in a pooling layer (*blue*), which results in a higher level of complexity for the next convolution. After the last pooling layer, all filters are flattened to a single layer that passes some fully connected layers (like a basic feedforward network, Fig. 4a) to finally come to a predicted output, where the predicted output class acronym *DCM* stands for dilated cardiomyopathy, *HCM* for hypertrophic cardiomyopathy and *RCM* for restrictive cardiomyopathy

Convolutional neural networks (CNNs) are an evolution of the traditional neural networks (Fig. 4a) and currently the most popular DL models. Inspired by neural networks in the visual cortex in mammals, CNNs consist of filters to detect objects of different levels of complexity (Fig. 5). CNNs are not highly dependent on object location and scale, and require less computational power.

#### Autoencoders

Autoencoders are artificial neural networks that learn the representation or encoding of data, focused on dimensional reduction and filtering of noise. The networks can be used to compress data or extract features that are used in a classifier, such as a support vector machine. Although the architecture of autoencoders may resemble supervised neural networks, no ground truth labels are involved and the method is considered an unsupervised DL technique.

#### Recurrent neural networks

Recurrent neural networks can be used to learn about and predict sequential data, such as ECG, continuous blood pressure measurements and perfusion curves. Instead of the typical feedforward networks mentioned before, these networks do not handle data at a specific timeframe, such as a single image, but keep track of prior data and changes in data over time (Fig. 3d). In long short-term memory networks, the most widely used recurrent neural networks, a model state can remember cumulative information from previous input and output to better interpret the current input (Fig. 3e). Notably, mobile phone keyboards represent a clear-cut example of these networks. They can use contextual information from previous words to better predict the next desired word. In cardiology, recurrent neural networks can be used to label intervals in ECGs, or to diagnose arrhythmias.

### Overfitting, cross-validation and data augmentation

It is of great importance to recognise that model training and optimisation are not indefinite processes in ML. At a certain point, after a number of training and validation iterations, any model may begin to demonstrate almost perfect predictions on the training data, while its performance on the validation and test sets rapidly decreases. This indicates that the model has memorised the training samples, rather than learned relevant features and relations between them for further generalisation. This phenomenon is formally known as overfitting, and several manoeuvres can help prevent it. One is adding more training cases, although it is not always possible. Another method is to add training data through controlled and subtle modifications of the existing one through, for example, image rotation and zooming. This procedure is known as data augmentation. Finally, it is also possible to apply cross-validation, which works by randomly parcellating data for training and validation iterations to increase the variety of examples presented to the model while making effective use of all cases for both purposes [19].

## Current and near-future cardiovascular applications

### Automation

Classification algorithms are already applied in several software packages for the pre-processing of echocardiographic imaging into specific views and image orientations, to improve automation and time efficiency of the human interpreter [20, 21]. A more recent implementation is image segmentation. For example, cardiac chambers can be delineated automatically and values such as ejection fraction or longitudinal strain measurements can be calculated (Fig. 5; [22]). Some complete solutions for echocardiography are already available [23], allowing less human time investment and upscaling of automated image analysis, and implementation into clinical practice. DL-based segmentation of cardiac CT and MRI assessments has also been developed [24–27]. Results were shown to be on par with human experts [28].

### Risk stratification and prediction

DL-based classification models are currently used in screening programs for detection and classification of diseases. For example, new DL-based programs are being used for the detection of malignant lung nodules on CT images [29]. Similar screening methods could be used to predict the presence, or absence, of coronary artery disease (CAD). For instance, CNNs have been developed for the automated coronary artery calcium scoring on CT scans to determine cardiovascular risk [30, 31].

ML has offered the possibility to predict PET myocardial perfusion imaging results based on the integration of simple data better than with traditional statistical models [32]. This suggests that selection of patients to be referred for specialised and costly imaging may be optimised with the help of AI.

Recently, an ML method based on random forest classification has been applied to integrate genetic and clinical information to improve prediction of CAD over conventional risk factors [33].

### Diagnostics

Various CNNs as well as recurrent neural networks have been used to interpret ECG signals and detect arrhythmias, to analyse fluctuations in the ECG over time, and to relate these fluctuations to the incidence of cardiovascular events [34–36].

In image analysis, automated calculation of FFR from CCTA images has been used to identify patients with functional coronary artery stenosis [10, 37]. Similarly, DL-based and support vector machine algorithms have been used to detect functionally significant CAD on SPECT myocardial perfusion imaging [38]. Furthermore, classification algorithms have been applied for automated detection of aneurysms [39] and thrombosis [40].

Diastolic dysfunction of the left ventricle has represented an elusive concept, and the exact definition and diagnostic criteria have been much debated in recent years. Clustering algorithms have been applied to echocardiographic parameters of diastolic function, which have identified two distinct groups with improved prediction of event-free survival over conventional classification [41].

### Treatment response and prognosis

ML algorithms might have additional value in predicting treatment response, thereby identifying subgroups of patients that can benefit from specific therapies. Recently, an unsupervised ML clustering algorithm of clinical parameters and imaging data has shown that responders on cardiac resynchronisation therapy may be predicted by clustering them into subgroups [42]. A support vector machine approach has been used to distinguish between different phenotypes of coronary plaque, which could help identify high-risk plaques for which therapy should be intensified in order to prevent major adverse cardiac events [32, 43]. Clustering analyses have been used on demographic, clinical, laboratory, imaging and medication data from heart failure patients to identify different subgroups thereof [44, 45]. These subgroups were shown to develop different clinical outcomes and response to treatment. In conjunction with support vector machines and random forests, clustering may facilitate further development of precision medicine, focusing on an ontology-based classification approach [46–48].

Electronic health records are an important source of (unstructured) data that has great potential for the application of predictive ML-based models. Three types of DL models trained on unstructured electronic health record data have been shown to achieve high accuracy for tasks such as predicting in-hospital mortality and readmission [49]. Furthermore, when the algorithm predicted a high chance of an adverse outcome, the authors demonstrated that neural networks were able to identify and highlight relevant information from the patient’s chart that was used to predict this adverse outcome. For clinicians, this could be a useful tool that serves as a safety net to not overlook important information.

Most of today’s DL models have not been designed for prognostics, and lack the ability to make predictions of the estimated time to an adverse event. A Cox proportional hazard model equivalent has been built and demonstrated by the Cox-NNet [50], but no applications in the cardiovascular domain have been published so far.

## Limitations

AI techniques may be used to solve more complex problems than traditional statistics, but the application of these techniques is accordingly more complex. Besides a basic understanding of the techniques, it requires sufficient programming skills to set up a model, and the knowledge to interpret output from diagnostic tools to assess model performance and prevent model overfitting.

An important assessment that is finding its way along the application of AI in clinical research problems is the estimation of a problem’s complexity. Even though it is true that ML can perform better that traditional statistics on complex data, it is equally correct to recognise that traditional linear models are robust and may suffice for many analyses. The identification of datasets and objectives that can benefit from AI implementation is a growing necessity in modern analytical workflows.

Another relative limitation in ML-based AI is that best-performing models are continuously revised and improved, and today’s state-of-the-art architectures may become obsolete within years or even months. For instance, the CNN, which today is probably applied most often to single-frame data, was criticised and proposed to be replaced by the more advanced capsule network [51] by the same researchers that triggered the advancement of DL by winning the ImageNet competition using a CNN [6]. Capsule networks are less prone to false-positive classifications because they develop knowledge about lower-level objects’ relative locations and orientations. They are also less prone to false negatives because they handle better orientation variations, such as rotation or flipping of classified objects. This approach is sufficiently more complex to implement than conventional CNNs and will, therefore, take time to be implemented on a larger scale.

Full AI expansion is currently limited by data accessibility and structure. To make use of expanding available data through DL algorithms, dataset standardisation and combination will be paramount. Furthermore, missing values in structural data constitute an additional challenge that clinicians must presently have in mind to manage and continue expanding their datasets. Better regulatory mechanisms that favour data sharing and security have to be established.

## Future perspectives

In selected areas, AI has great potential to be used for clinical support tools, already performing on a par or even better than human experts. Implementations in imaging and ECG systems can be expected in hospitals shortly, improving reproducibility and accuracy of measurements, diagnoses and treatment decisions. ML models using imaging or ECG data to predict coronary heart disease will help prevention of unnecessary cardiac catheterisations and admissions for acute coronary syndrome. Implementation of tools to better detect arrhythmias and more subtle ECG abnormalities can facilitate better risk stratification and remote patient monitoring using smartphone apps [52]. Application of predictive models for treatment response will allow for patient-tailored therapy.

AI technology has already been integrated in patient electronic record systems, providing options for enhancing the workflow, and providing early warning systems for real-time evaluation to identify patients at a high risk of experiencing adverse events. Clinical ML-based decision support tools are being widely investigated in studies and in some cases are already integrated in electronic health record systems. As already seen on mobile devices and in online mail systems, implementation of speech recognition and word prediction tools in electronic health record systems will reduce the time spent by doctors on administrative tasks, resulting in more effective time to address the treatment of patients.

Eventually, the potential for ML models to predict any patient’s outcome based on the information from an almost limitless source of data from similar patients should provide very accurate predictions of the development of cardiovascular disease that can be prevented with targeted interventions, prevent the incidence of unexpected adverse events in hospitals and provide tailored therapy that will greatly improve patient outcome.

## Conclusions

Modern ML-based AI is proving to be capable of solving complex problems and saving time by automating time-consuming human operations. Implementations in cardiovascular research clinical practice are gradually taking off and are expected to ultimately cover the whole range of risk stratification, diagnosis, treatment and prognosis of cardiovascular disease.

Near-future implementations of AI will provide clinicians with diagnostic tools, clinical decision systems and greatly enhanced workflows in electronic health records, reducing costs in healthcare, improving the level of patient care and enabling doctors to focus more on their actual responsibility, treating patients.

Acknowledgements

The Research Project CVON-AI (2018B017) is financed by the PPP Allowance made available by Top Sector Life Sciences & Health to the Nederlandse Hartstichting to stimulate public-private partnerships. This work reflects only the author’s view, not that of the funders. Stichting LSH-TKI or Hartstichting or the Ministry of Economic Affairs is not responsible for any use that may be made of the information it contains.
